# Pivotal role of human stearoyl-CoA desaturases (SCD1 and 5) in breast cancer progression: oleic acid-based effect of SCD1 on cell migration and a novel pro-cell survival role for SCD5

**DOI:** 10.18632/oncotarget.25273

**Published:** 2018-05-11

**Authors:** Cristiana Angelucci, Alessio D'Alessio, Fortunata Iacopino, Gabriella Proietti, Alba Di Leone, Riccardo Masetti, Gigliola Sica

**Affiliations:** ^1^ Istituto di Istologia e Embriologia, Università Cattolica del Sacro Cuore, Roma, Italia; ^2^ Unità Operativa di Chirurgia Senologica, Università Cattolica Del Sacro Cuore, Fondazione Policlinico Universitario A. Gemelli, Roma, Italia

**Keywords:** breast cancer cells, cancer-associated fibroblasts, stearoyl-CoA desaturase, cell migration, necrosis

## Abstract

The influence of cell membrane fluidity on cancer progression has been established in different solid tumors. We previously reported that “cancer-associated fibroblasts” (CAFs) induced epithelial-mesenchymal transition and increased cell membrane fluidity and migration in poorly (MCF-7) and highly invasive (MDA-MB-231) breast cancer cells. We also found that the membrane fluidity regulating enzyme stearoyl-CoA desaturase 1 (SCD1) was upregulated in tumor cells co-cultured with CAFs and established its essential role for both intrinsic and CAF-driven tumor cell motility. Here, we further explored the mechanisms involved in the SCD1-based modulation of breast cancer cell migration and investigated the role of the other human SCD isoform, SCD5. We showed that the addition of oleic acid, the main SCD1 product, nullified the inhibitory effects produced on MCF-7 and MDA-MB-231 cell migration by SCD1 depletion (pharmacological or siRNA-based). Conversely, SCD5 seemed not involved in the regulation of cancer cell motility. Interestingly, a clear induction of necrosis was observed as a result of the depletion of SCD5 in MCF-7 cells, where the expression of SCD5 was found to be upregulated by CAFs. The necrotic effect was rescued by a 48-h treatment of cells with oleic acid. These results provide further insights in understanding the role of SCD1 in both intrinsic and CAF-stimulated mammary tumor cell migration, unveiling the metabolic basis of this desaturase-triggered effect. Moreover, our data suggest the ability of CAFs to promote the maintenance of tumor cell survival by the induction of SCD5 levels.

## INTRODUCTION

Metastatic progression is mostly responsible for the fatal outcome of human carcinomas and it is ultimately driven by an improved cancer cell migration ability. In this respect, the key role played by the interaction between the epithelial tumor cells and the stromal microenvironment has been established in different malignancies, with a stroma-triggered induction of tumor cell migration/invasiveness being reported [1–4]. Cell membrane fluidity represents a crucial element in regulating various aspect of cell biology. Indeed, alterations in the balance of saturated fatty acids (SFAs) and monounsaturated fatty acids (MUFAs) of the lipid bilayer can influence a wide variety of cell functions, including cell motility [5–9].

Stearoyl-CoA desaturase (SCD) is an endoplasmic reticulum-resident D9 desaturase which regulates membrane fluidity by the conversion of endogenous and exogenous SFAs into MUFAs. Two SCD isoforms (SCD1 and SCD5) have been identified in humans, whereas four desaturases (SCD1-SCD4) exist in mice, which, despite different tissue distribution patterns, share the same enzymatic function [10]. Human SCD1, the prevalent isoform, is ubiquitously expressed among tissues. High SCD1 levels are correlated with metabolic disorders such as obesity and insulin resistance but, more recently, this desaturase has been identified as a key regulator of cell growth, programmed cell death and carcinogenesis [11, 12]. SCD1 overexpression has been reported in human cancers, carcinogen-induced tumors and virus-transformed cells, resulting in an enhancement of membrane fluidity [13–15]. In mammary cancer cells, SCD1 pharmacological inactivation or silencing has been found to decrease tumor cell proliferation and to inhibit glucose-mediated lipogenesis [16, 17]. Increased tumor cell membrane fluidity has also been shown to promote malignant cell migration ability [9]. We previously reported an overall pro-invasive effect exerted on mammary cancer cells by the main elements of the tumor stroma, the so-called “cancer-associated fibroblasts” (CAFs), as they were able to induce “epithelial-mesenchymal transition” (EMT) and an increase in membrane fluidity, as well as in migration speed and directness [18]. We also demonstrated a critical contribution of CAF-released soluble factors such as hepatocyte growth factor (HGF), basic fibroblast growth factor and transforming growth factor-β in promoting tumor cell migration speed [19]. Consistent with the induction of a more loose packing of the membrane lipid bilayer in mammary cancer cells, we previously showed the ability of CAFs to promote SCD1 upregulation and demonstrated the key role played by the desaturase in both intrinsic and CAF-prompted tumor cell migration. In fact, we found that either siRNA-mediated or pharmacological inhibition of SCD1 impaired tumor cell migration [19].

The second isoform of SCD identified in humans is SCD5, which is unique to primates and highly expressed in brain and pancreas [10]. Unlike SCD1, few information is available about the physiological role of SCD5 as well as on its involvement in pathological processes [20, 21].

The aim of the present study was to gain further insight into the mechanisms responsible for the SCD1-mediated regulation of breast cancer cell migration [19], as well as the assessment of the possible role played by SCD5 in this setting. In the poorly invasive or highly metastatic breast cancer cell lines (MCF-7 and MDA-MB-231, respectively) that we used in our previous studies, we demonstrated that the inhibitory effect produced by SCD1 depletion on the intrinsic and CAF-promoted cancer cell migration was ascribable to the scarcity of oleic acid, the enzyme prevalent metabolic product. On the contrary, SCD5 appeared not to be significantly involved in the regulation of migration of tumor cells but needed to protect them from necrotic insults. The observed increase in SCD5 levels induced by CAFs outlines a further role of these stromal cells in breast cancer progression by the promotion of tumor cell survival.

## RESULTS

### CAFs induce SCD5 expression in tumor cells

**Figure 1:**
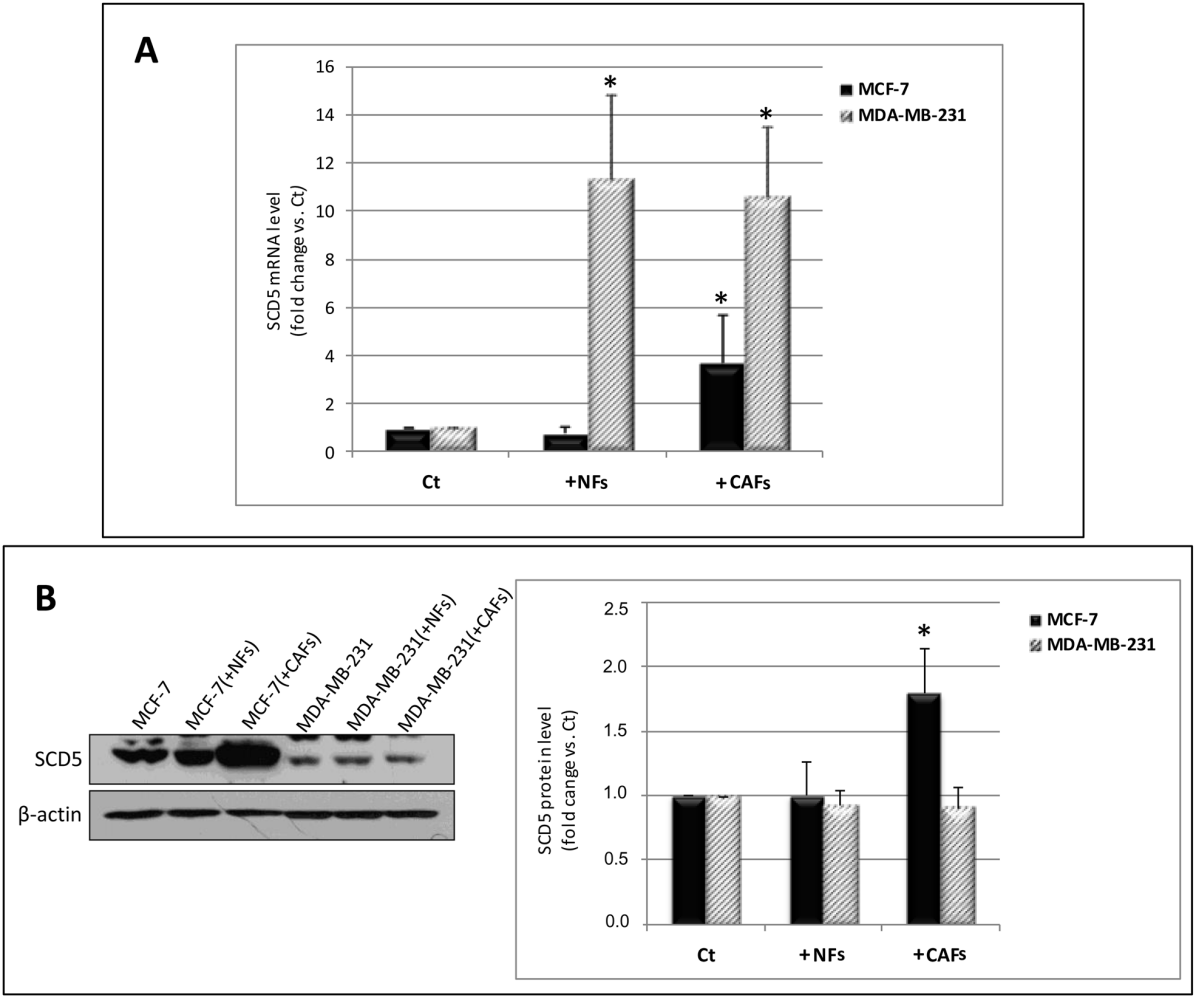
CAFs differentially affect SCD5 gene and protein expression in MCF-7 and MDA-MB-231 cells **(A)** Increase in SCD5 mRNA level in MCF-7 cells as a result of 6-day co-culture with CAFs and in MDA-MB-231 cells due to the interaction with NFs or CAFs. Tumor cells were magnetically isolated from fibroblasts, total RNA was extracted and qRT-PCR analysis was performed. The relative fold change in mRNA expression was calculated using the 2−ΔΔCt method. The mean of ΔCt values for SCD5 amplicon was normalized to that of β-actin and compared with control (MCF-7 or MDA-MB-231 cell monocultures, Ct). **(B)** Increase in SCD5 protein level in MCF-7 cells as a result of 6-day co-culture with CAFs. The same treatment did not significantly affect SCD5 protein expression in MDA-MB-231 cells. Tumor cells were magnetically isolated from fibroblasts and whole cell lysates subjected to Western blot analysis using the indicated anti-SCD5 antibody. A representative blot is shown on the left. Densitometric quantification (Quantity One quantitation software, Bio-Rad) is shown on the right. Data (arbitrary densitometric units) were normalized to β-actin and expressed as relative fold increase over control (Ct). (A, B) The values of mean + SD from three different co-culture experiments were presented. ^*^p<0.05 vs Ct, Student's t test.

The influence of CAFs was also investigated on SCD5 mRNA and protein levels in tumor cells. A significant increase in SCD5 transcript levels was found in MCF-7 cells co-cultured with CAFs (Figure 1A). The same effect was observed as for SCD5 protein expression (Figure 1B). In MDA-MB-231 cells, a strong increase in the desaturase mRNA levels was observed as an effect of the interaction with both NFs and CAFs (Figure 1A) which, however, did not match a corresponding protein upregulation (Figure 1B).

### Oleic acid addition rescues the inhibitory effect induced by SCD1 depletion on tumor cell migration

**Figure 2:**
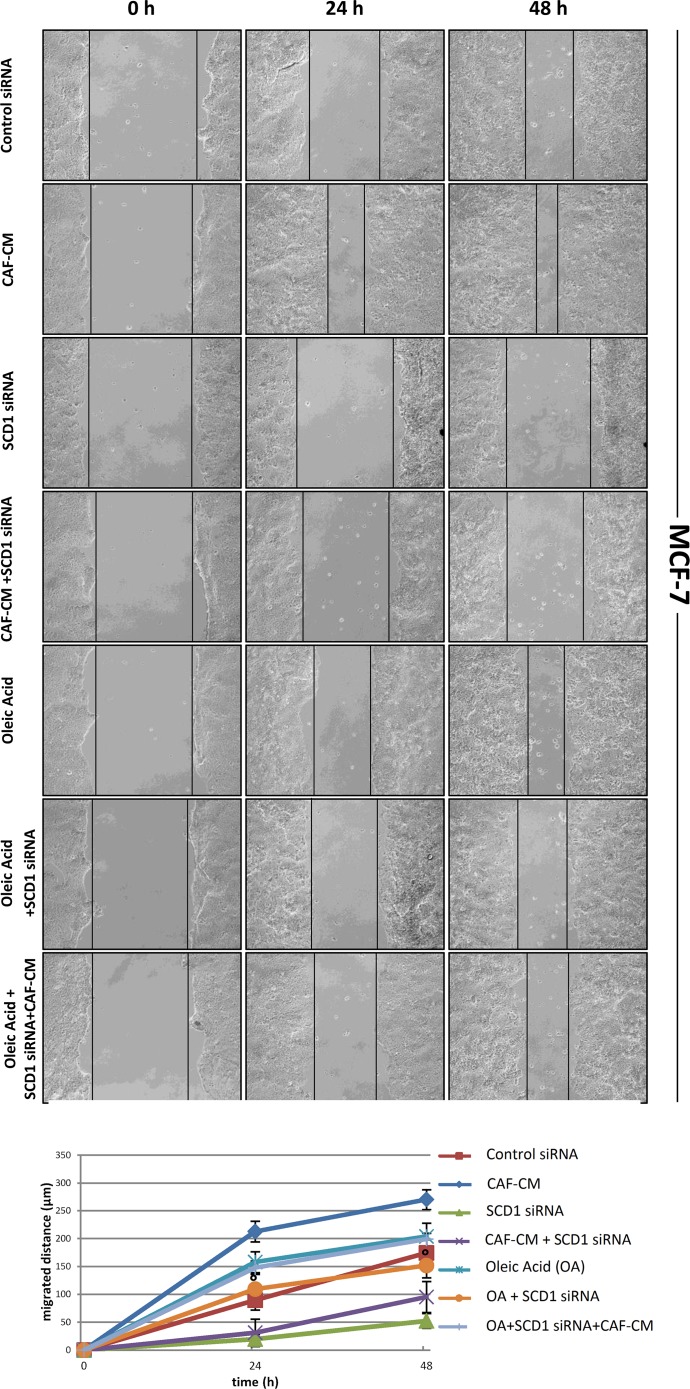
Oleic acid restores the original migration ability in poorly invasive tumor cells silenced for SCD1 by siRNA MCF-7 cells underwent a transient SCD1 knockdown by a 72-h transfection with 60 pmol of SCD1 siRNA oligos. Another series of cells was transfected with non-targeting siRNA oligos (control siRNA). The following treatments were then added to SCD1-depleted tumor cells (for 0, 24 and 48 h, until the evaluation of their migration): (a) CAF-CM, (b) 10 μM oleic acid, (c) CAF-CM plus 10 μM oleic acid. The cell migration activity was analyzed by a wound healing assay on subconfluence cells grown into 6-well plates. The gaps (5 randomly selected points per wound) were photographed at 0, 24 and 48 h after wounding under an inverted microscope (magnification, 100x) and the distance migrated by the cells was measured at the reference points by an image-processing software (ImageJ, version 1.49; imagej.nih.gov/ij/). Values in the graph are the mean + SD of three independent experiments (two sets of culture dishes for each condition). °p<0.001 vs SCD1 siRNA, Student's t test.

**Figure 3:**
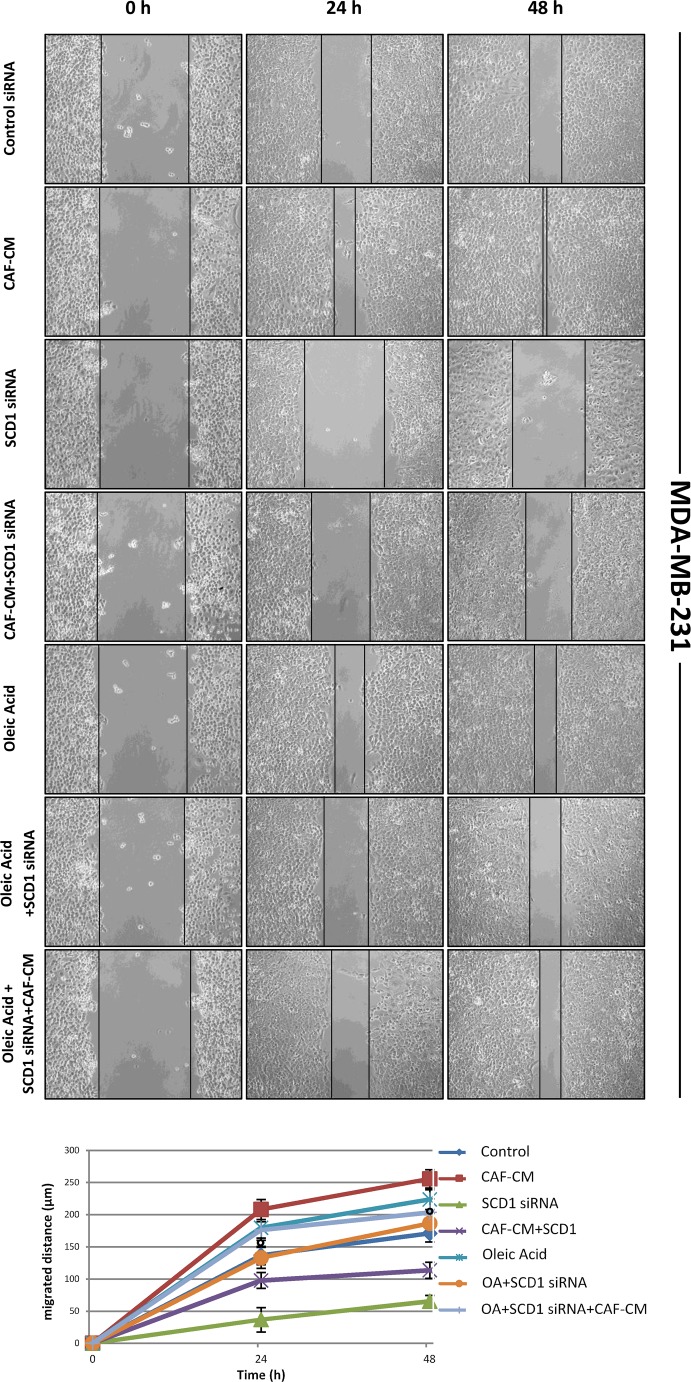
Oleic acid restores the original migration ability in highly invasive tumor cells silenced for SCD1 by siRNA MDA-MB-231 cells underwent a transient SCD1 knockdown by a 72-h transfection with 60 pmol of SCD1 siRNA oligos. Another series of cells was transfected with non-targeting siRNA oligos (control siRNA). The following treatments were then added to SCD1-depleted tumor cells (for 0, 24 and 48 h, until the evaluation of their migration): (a) CAF-CM, (b) 10 μM oleic acid, (c) CAF-CM plus 10 μM oleic acid. The cell migration activity was analyzed by a wound healing assay on subconfluence cells grown into 6-well plates. The gaps (5 randomly selected points per wound) were photographed at 0, 24 and 48 h after wounding under an inverted microscope (magnification, 100x) and the distance migrated by the cells was measured at the reference points by an image-processing software (ImageJ, version 1.49; imagej.nih.gov/ij/). Values in the graph are the mean + SD of three independent experiments (two sets of culture dishes for each condition). °p<0.001 vs SCD1 siRNA, Student's t test.

**Figure 4:**
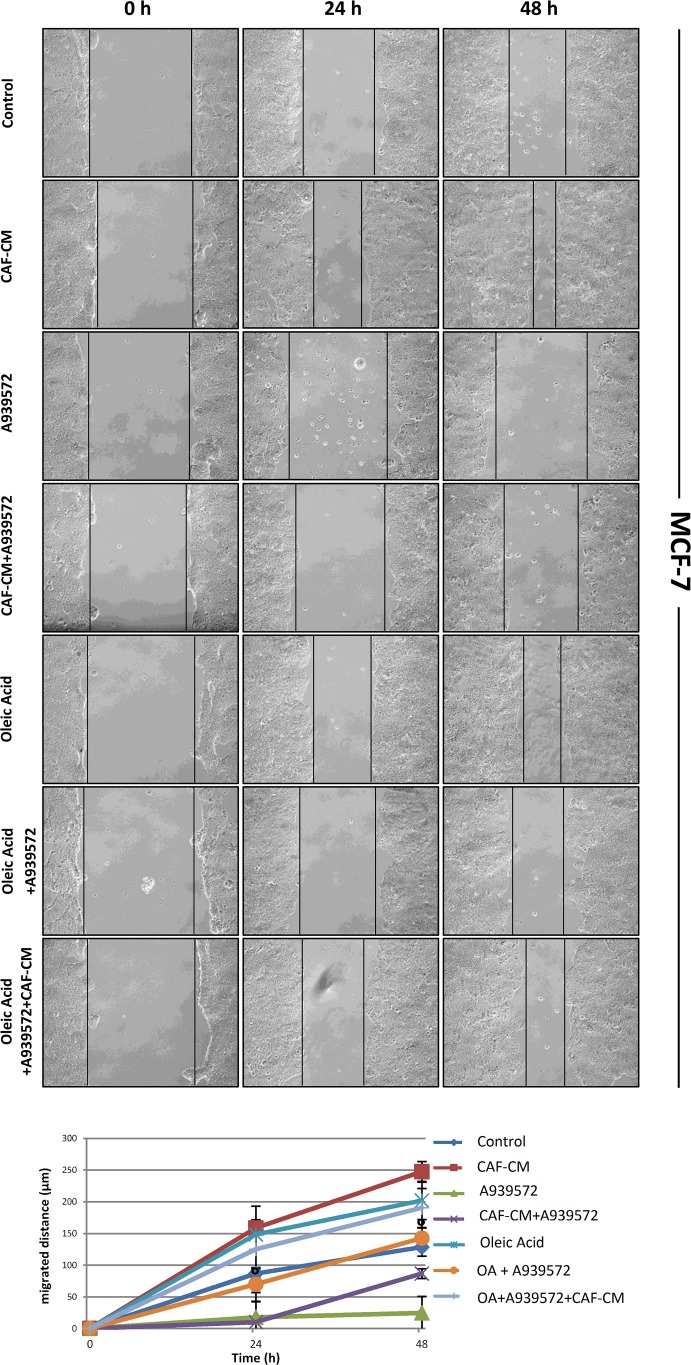
Oleic acid restores the original migration ability in SCD1 pharmacologically inhibited poorly invasive tumor cells SCD1 inhibition was obtained by treating MCF-7 cells for 24 and 48 h with the small molecule inhibitor of SCD1, A939572 (1 μM). As well as for the experiments with siRNA inhibition, also the SCD1 pharmacologically inhibited cells were exposed to: (a) CAF-CM, (b) 10 μM oleic acid, (c) CAF-CM plus 10 μM oleic acid. The cell migration activity was analyzed by a wound healing assay on subconfluence cells grown into 6-well plates. The gaps (5 randomly selected points per wound) were photographed at 0, 24 and 48 h after wounding under an inverted microscope (magnification, 100x) and the distance migrated by the cells was measured at the reference points by an image-processing software (ImageJ, version 1.49; imagej.nih.gov/ij/). Values in the graph are the mean + SD of three independent experiments (two sets of culture dishes for each condition). °p<0.001 vs A939572, Student's t test.

**Figure 5:**
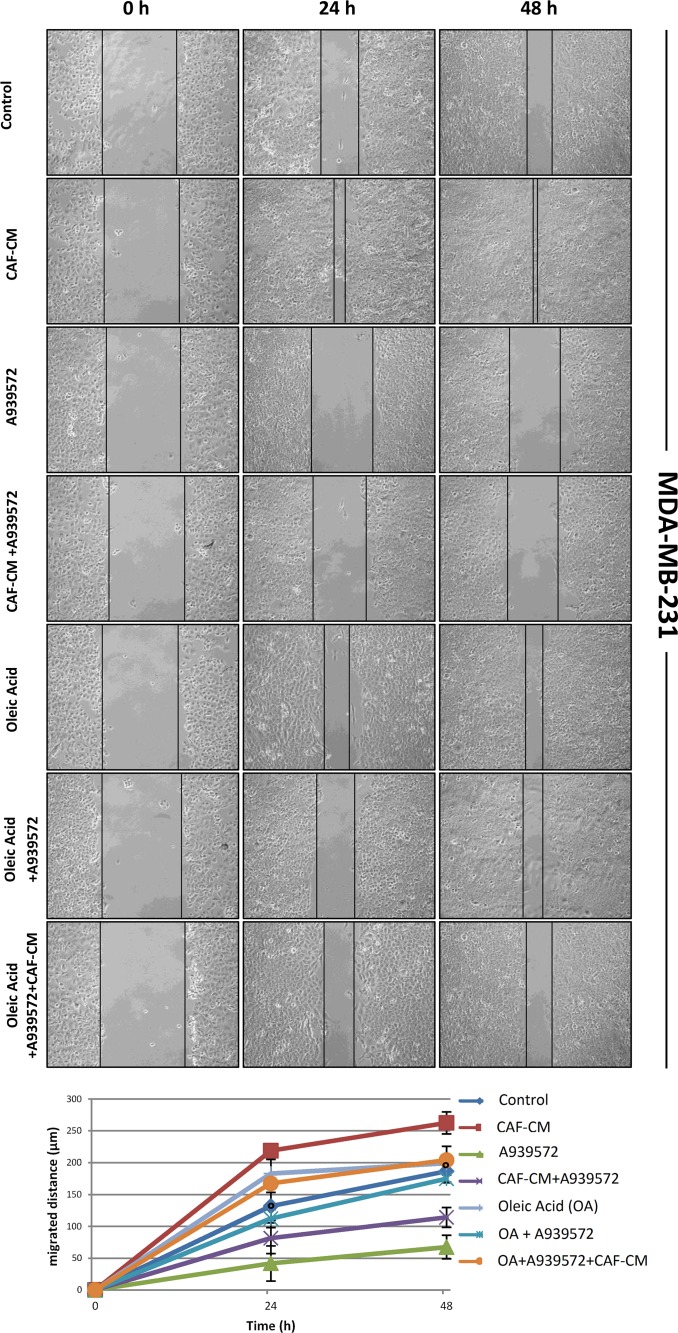
Oleic acid restores the original migration ability in SCD1 pharmacologically inhibited highly invasive tumor cells SCD1 inhibition was obtained by treating MDA-MB-231 cells for 24 and 48 h with the small molecule inhibitor of SCD1, A939572 (1 μM). As well as for the experiments with siRNA inhibition, also the SCD1 pharmacologically inhibited cells were exposed to: (a) CAF-CM, (b) 10 μM oleic acid, (c) CAF-CM plus 10 μM oleic acid. The cell migration activity was analyzed by a wound healing assay on subconfluence cells grown into 6-well plates. The gaps (5 randomly selected points per wound) were photographed at 0, 24 and 48 h after wounding under an inverted microscope (magnification, 100x) and the distance migrated by the cells was measured at the reference points by an image-processing software (ImageJ, version 1.49; imagej.nih.gov/ij/). Values in the graph are the mean + SD of three independent experiments (two sets of culture dishes for each condition). °p<0.001 vs A939572, Student's t test.

Since we recently demonstrated that pharmacological or genetic inactivation of SCD1 resulted in a significant inhibition of both intrinsic and CAF-promoted MCF-7 and MDA-MB-231 cell migration [19], here we sought to address whether this effect was due to the deficiency in oleic acid, the main product of SCD activity. The addition of 10 μM oleic acid to the culture medium appeared to provide a certain migratory stimulus in MCF-7 and MDA-MB-231 cells after 24 and 48 h (Figures 2-5) and, more noteworthy, was able to restore their original migration ability which was weakened by both SCD1 siRNA-depletion (Figures 2 and 3) or pharmacological inhibition (Figures 4 and 5) (p<0.001). These experiments also confirmed the stimulatory activity of the CAF-CM on tumor cell migration as well as the suppression of this effect by SCD1 inhibition (Figures 2-5). Finally, the addition of oleic acid to SCD1-inhibited tumor cells maintained in the CAF-CM restored a migratory ability comparable to that of the cells treated with oleic acid only (Figures 2-5).

### SCD5 does not affect MDA-MB-231 cell migration ability

**Figure 6:**
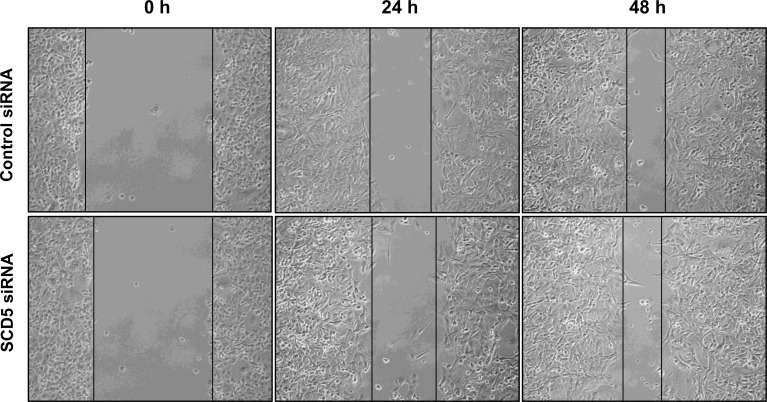
SCD5 does not influence migration of highly invasive mammary tumor cells MDA-MB-231 cells underwent a transient SCD5 knockdown by a 72-h transfection with 60 pmol of SCD1 siRNA oligos. The cell migration activity was analyzed by a wound healing assay on subconfluence cells grown into 6-well plates. The gaps (5 randomly selected points per wound) were photographed at 0, 24 and 48 h after wounding under an inverted microscope (magnification, 100 x) and the distance migrated by the cells was measured at the reference points by an image-processing software (ImageJ, version 1.49; imagej.nih.gov/ij/). Control siRNA (MCF-7 transfected with non-targeting siRNA oligos). Three independent experiments were performed with two sets of culture dishes for each condition.

The same wound healing experiments as those above described to evaluate the effect of SCD1 depletion by siRNA and oleic acid treatment on tumor cell migration were carried out to assess the potential role of SCD5. In the two cell lines, the effect produced by the inhibition of SCD5 was different. In MCF-7 cells, starting from the first hours after SCD5 silencing a massive detachment of the cells was observed (not shown). On the other hand, MDA-MB-231 cell migration was not significantly affected by SCD5 depletion (Figure 6).

### SCD5 activity is needed to preserve MCF-7 cell viability without affecting adhesion molecule expression

**Figure 7:**
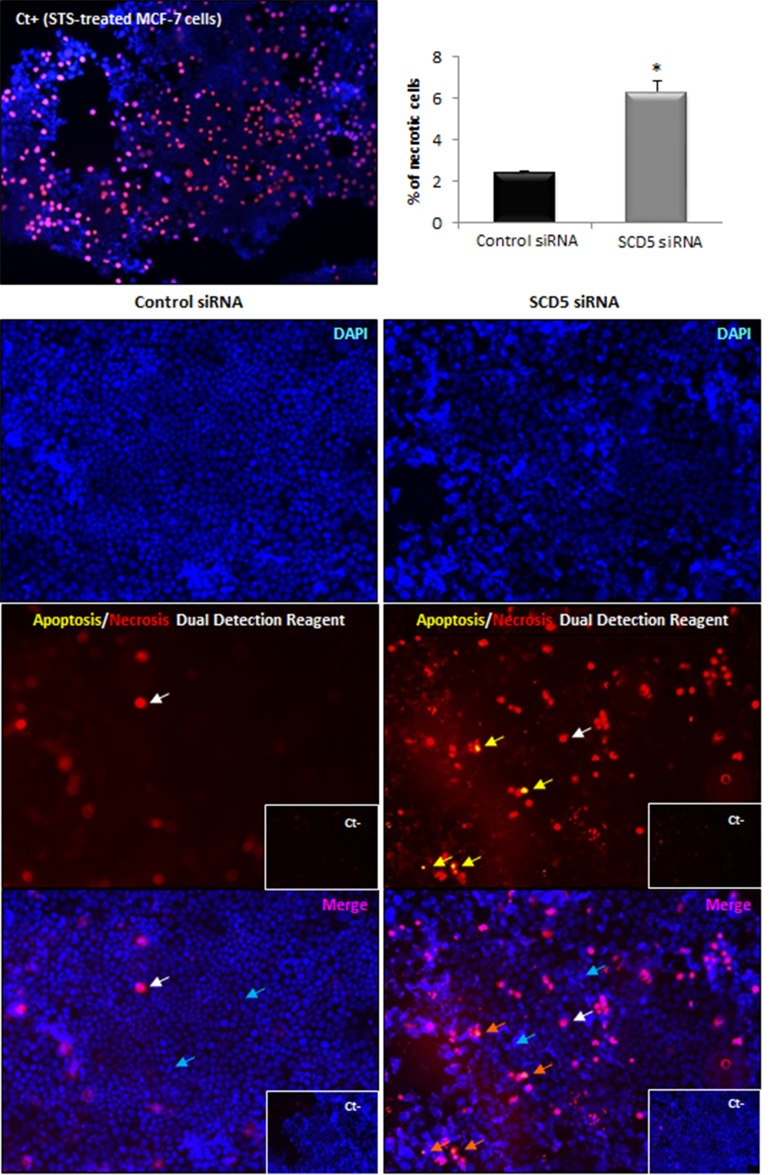
SCD5 knockdown promotes necrotic cell death of MCF-7 cells Apoptosis/necrosis analysis was performed by an “Apoptosis/Necrosis Detection Kit” (ENZO Life Sciences) on MCF-7 cells that underwent a SCD5 knockdown by a 72-h transient transfection with 60 pmol of SCD5 siRNA oligos. Cells were washed with PBS and incubated with apoptosis (annexin V-EnzoGold)/necrosis (membrane impermeable DNA intercalating dye-7-AAD) dual detection reagent, according to the manufacturer's protocol and observed under an Olympus BX53 fluorescent microscope. The concurrent cell staining with annexin V (yellow) and 7-AAD (red) identifies cells in late apoptosis (yellow arrows in the central right panel, orange arrows in the bottom right panel). Staining with the only necrosis red dye (red in the central panels and pink in the bottom merged images, white arrows) detects necrotic cells. Nuclear counterstaining was performed with the blue fluorescent dye DAPI (turquoise arrow). Control siRNA (MCF-7 transfected with non-targeting siRNA oligos). Negative controls (Ct-) were obtained by omitting incubation with dual detection reagent. MCF-7 cells treated with Staurosporine (STS, 2 μM) were used as positive control (Ct+) for necrosis [37]. Images (magnification, 200x) were captured with ISCapture software (Tucsen Photonics) and are representative of three independent experiments. Data are shown as mean + SD of three independent experiments. ^*^p<0.001 vs control siRNA, Student's t test.

Given the SCD5 depletion-associated detachment of the MCF-7 cell monolayer, we sought to assess the possible influence of the enzyme in the maintenance of tumor cell viability. Thus, MCF-7 cells that underwent SCD5 siRNA transfection and MCF-7 cells transfected with non-targeting siRNA (control) were subjected to an apoptosis/necrosis assay. As shown in Figure 7, this analysis highlighted the involvement of SCD5 in the mechanisms responsible for tumor cell survival. Indeed, SCD5-silenced cells displayed a clear induction of necrotic death with respect to control cells (2.6 fold increase, p<0.001), while the desaturase depletion did not give rise to any apoptotic signs (Figure 7). In more detail, three populations of cells were detectable: (1) cells that were viable and not apoptotic or necrotic (annexin V/Cy3- and 7-AAD-negative), (2) cells undergoing late-stage apoptosis (annexin V/Cy3- and 7-AAD-positive), and (3) cells undergoing necrosis (annexin V/Cy3-negative and 7-AAD-positive).

Interestingly, the simultaneous knockdown of SCD1 and 5 in MCF-7 and MDA-MB-231 cells did not induce a significant worsening of cell viability in the former model with respect to the cytotoxic effect produced by the single SCD5 silencing (Supplementary Figure 2). As regards MDA-MB-231, double SCD1/SCD5 siRNA transfection seemed not to affect cell viability, as observed in the cells that underwent the single SCD1- or SCD5-silencing (Supplementary Figure 3).

**Figure 8:**
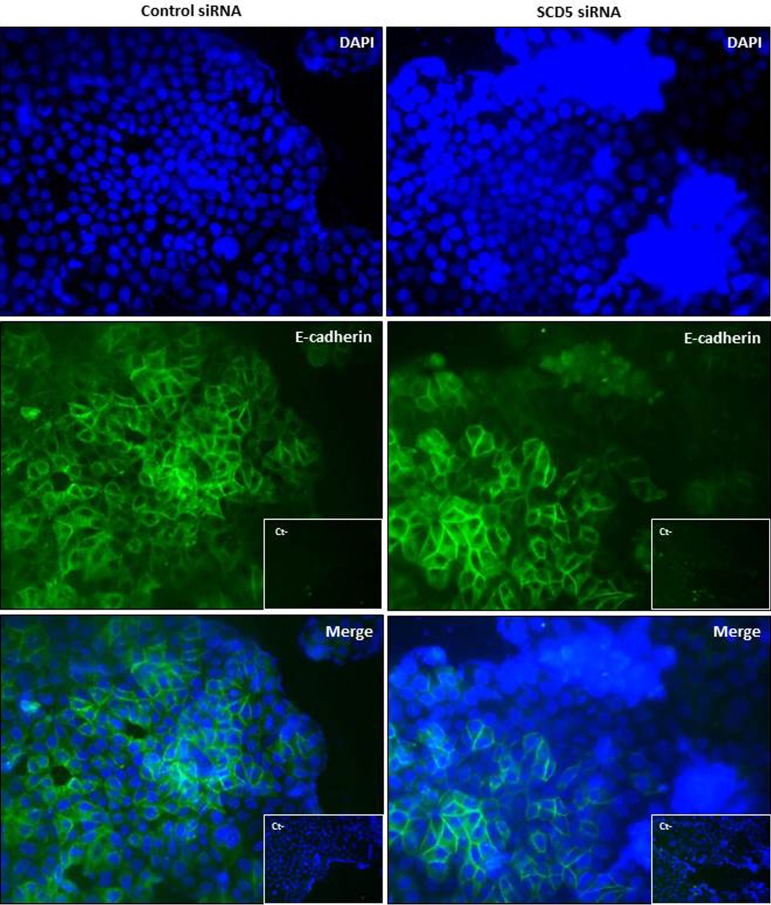
SCD5 knockdown does not affect E-cadherin expression in low invasive mammary tumor cells Immunofluorescence analysis of E-cadherin expression (green) on MCF-7 cells, that underwent a SCD5 knockdown by a 72-h transient transfection with 60 pmol of SCD5 siRNA oligos, reveals no substantial differences in the adhesion molecule expression with respect to control (MCF-7 cells transfected with non-targeting siRNA oligos). DAPI-stained nuclei appear in blue (magnification, 400x).

Against the observed detachment of MCF-7 cells as a result of SCD5 depletion, immunofluorescence analysis did not reveal any significant change in the expression of the epithelial cell-cell adhesion protein E-cadherin (Figure 8) or a *de novo* expression of the mesenchymal N-cadherin (not shown).

### Oleic acid supply counterbalances the necrotic effect induced by SCD5 depletion

**Figure 9:**
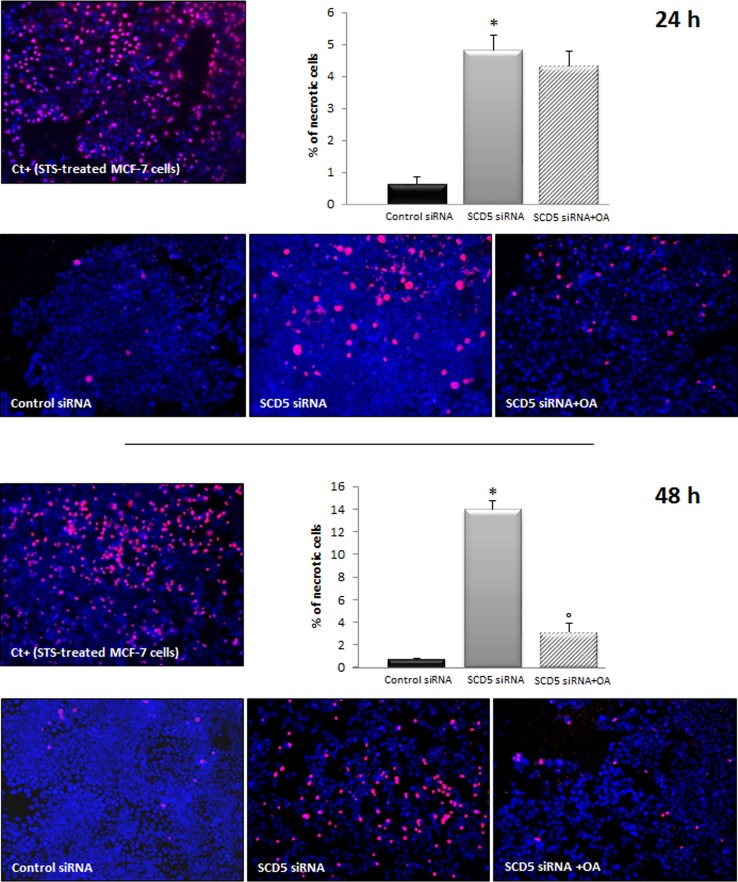
Oleic acid rescues MCF-7 cells from necrotic cell death induced by SCD5 knockdown Apoptosis/necrosis analysis was performed by an “Apoptosis/Necrosis Detection Kit” (ENZO Life Sciences) on MCF-7 cells that underwent a SCD5 knockdown by a 72-h transient transfection with 60 pmol of SCD5 siRNA oligos. Ten μM oleic acid (OA) was added to the culture medium of SCD5 siRNA silenced cells and the effect of the treatments on cell viability evaluated after 24 and 48 h. MCF-7 cells treated with Staurosporine (STS, 2 μM) were used as positive control (Ct+) for necrosis [37]. Images (magnification, 200x) were captured with ISCapture software (Tucsen Photonics) and are representative of three independent experiments. Data are shown as mean + SD of three independent experiments. ^*^p<0.001 vs control siRNA, ° p<0.001 vs SCD5 siRNA, Student's t test.

As we found that the addition of oleic acid was able to rescue MCF-7 cell migration from the inhibitory effect induced by SCD1 knockdown, we investigated the effect of oleic acid supplementation on the viability of SCD5-silenced MCF-7 cells. As above described (Figure 7), SCD5 depletion was able to significantly decrease MCF-7 cell viability. The addition of 10 μM oleic acid to the culture medium did not substantially modify this effect after 24 h, while, prolonging the treatment to 48 h, a significant improvement in cell viability was observed, as a marked reduction of necrotic cell death was evident in oleic acid-treated/SCD5-silenced cells with respect to the SCD5-inhibited ones (p<0.001; Figure 9).

## DISCUSSION

The contribution of the multifaced tumor stroma-derived signaling to breast cancer metastatic progression has been ascertained, but the molecular pathways underlying the acquisition of a more invasive phenotype in cancer cells are complex and still incompletely elucidated [22, 23].

We previously reported that CAFs, isolated from the stroma of mammary cancer specimens, induced EMT and an enhancement in cell membrane fluidity as well as in migration speed and directness in well- (MCF-7) and poorly-differentiated (MDA-MB-231) breast cancer cells [18]. In the same models, we also observed CAF-mediated upregulation of SCD1, a key regulator of membrane fluidity. Furthermore, we demonstrated the critical role of this desaturase in the mechanisms responsible for both intrinsic and CAF-promoted tumor cell migration that, indeed, was severely impaired by either SCD1 silencing or pharmacological inhibition [19]. In the present study, we deepened our previous findings by focusing more in detail on the mechanisms involved in the SCD1-based control of breast cancer cell migration and demonstrated an unpredicted role for the other human SCD isoform, SCD5, in the maintenance of tumor cell survival. In the two above described cell lines, we found that the inhibitory effect produced on tumor cell migration by SCD1 depletion is ascribable to the resulting deficiency of oleic acid, the main product of the desaturase enzymatic activity. Indeed, addition of exogenous oleic acid to the culture media of both low and highly invasive cancer cells overcomes the inhibitory effects produced by SCD1 blockade and restores their original migratory ability. This finding, consistently with our previous results [18, 19], suggests a metabolic basis of the CAF-mediated regulation of tumor cell motility and identifies SCD1-derived MUFAs, namely oleic acid, as the missing link in the axis CAFs/SCD1/cell migration. Targeted SCD1 inhibition has been reported to produce antitumor effects in different tumors. Mauvoisin D *et al* [24] observed a reduction in cell growth, ERK 1/2 activation and β-catenin nuclear translocation in SCD1-silenced breast cancer cells. A significant inhibition of cell proliferation, anchorage-independent growth and *in vivo* tumorigenesis was also described as a result of SCD1 depletion in human lung adenocarcinoma cells [25]. In the same cell type, it has been found that inhibition of this desaturase led to an impairment of Akt activation and a clear induction of apoptosis that could not be reversed by exogenously added oleic acid [25]. On the other hand, a metabolic basis of the antiproliferative effect triggered by SCD1 blockade has been reported in squamous cell carcinoma of the hypopharynx as the cell growth inhibitory effect produced by SCD1 depletion was found to be rescued by addition of BSA-conjugated oleic acid [26]. The effects produced by oleic acid seem, however, to be tissue-specific as in human esophageal cancer cells the MUFA reduced cell growth and migration through the upregulation of tumor suppressor genes such as p53, p21 and p27 [27]. Consistent with our findings, oleic acid treated breast cancer cells have been shown to undergo pro-tumor changes, as the increase in cell proliferation and survival through PI3K/Akt activation, whereas palmitic acid, the main SFA in mammalian cells, produced opposite effects [28]. Treatment of MDA-MB-231 cells with oleic acid has been previously described to promote their invasiveness through enhanced MMP-9 secretion. The same effects were not observed in the poorly invasive MCF-7 cells [29]. Unlike SCD1, the second human isoform of SCD, SCD5, seemed not to play a major role in the migratory ability of highly invasive breast cancer cells since its knockdown did not produce relevant variations in MDA-MB-231 cell migration. Accordingly, we found this desaturase levels to be upregulated through their interaction with CAFs in MCF-7 but not in MDA-MB-231 cells. Interestingly, SCD5 silencing in MCF-7 cells has revealed its involvement in the mechanisms responsible for tumor cell survival. In fact, against siRNA SCD5 depletion, an induction of necrotic cell death was detected in these latter cells without affecting adhesiveness, suggesting an alternative pathway leading to tumor progression based on the CAF ability to promote cell survival through SCD5 upregulation. These findings reveal an unexpected and unidentified function for SCD5. In fact, a pro-survival role was reported for SCD1 in lung cancer-initiating cells [30] as well as in human lung carcinoma or squamous cell carcinoma of the hypopharynx [26], but not for SCD5, whose potential role in human disease remains largely unknown. This desaturase is highly expressed in brain where it seems to play a key role in the mechanisms of cell growth and differentiation. In nervous cells the enzyme, through a deep deregulation of the epidermal growth factor and Wnt signaling promotes cell proliferation and inhibits neuronal differentiation [20]. The role of SCD5 in promoting mammary cancer cell survival is consistent with the incomplete response to neoadjuvant chemotherapy observed in breast cancer patients presenting significantly higher levels of the enzyme [31]. The authors hypothesized a predicting role for the peroxisome proliferator-activated receptor (PPAR), a transcription factor which is involved in tumor growth and whose activation leads to SCD5 gene expression, in affecting the response to taxane-based chemotherapy in breast cancer. Here, we found that 10 μM oleic acid supplementation rescued MCF-7 cells from necrotic cell death resulting from SCD5 knockdown. Radde BN et al have been recently reported that oleic acid (up to 25 μM) increases cell viability of both MCF-7 and MDA-MB-231 cells [32]. A proper balance between SFAs and unsaturated fatty acids, particularly MUFAs, is required for maintaining cell viability and homeostasis. SCD5 silencing, as previously reported for SCD1 [33], most likely determines a decrease in membrane oleic acid concentration and a parallel accumulation of SFAs. The membrane perturbing effect of increasing SFA concentration has been found to significantly reduce cell viability, even in MCF-7 cells, most likely *via* the induction of necrosis and cell lysis [34, 35]. In particular, the SFA palmitic acid has been found to act directly *via* endoplasmic reticulum (ER) to initiate a lipotoxic response [36]. The authors reported that the exogenously supplied SFA is rapidly embedded within lipid components of ER, impairing ER integrity. The higher sensitivity of MCF-7, with respect to MDA-MB-231 cells, to the ER stress may account for their susceptibility to the cytotoxic effect induced by SCD5 depletion.

The results presented here provide novel evidence that better define the role of SCD1 in the mechanisms underlying promotion of breast cancer cell migration by CAFs. Indeed, this study shows the existence of a direct relationship between different effects we previously reported to be induced by CAFs on mammary tumor cells, namely the increase in plasma membrane fluidity, the upregulation of SCD1 gene and protein levels and the induction of cell migration. Oleic acid has been here identified as the missing link in this SCD1-focused cascade of events which may ultimately lead to an increased tumor cell migration. These data, along with compelling previous reports demonstrating the significant involvement of SCD1 in tumor cell growth and survival, strengthen its role as a conceivable therapeutic target in this malignancy.

To our knowledge, the present study represents the first report demonstrating the survival-promoting role of SCD5 in breast cancer cells. As yet, little is known about the possible pathophysiological role of this other human SCD isoform in cancer and our findings encourage exploring its involvement in breast tumor progression with particular reference to the elucidation of the molecular mechanisms of tumor cell growth and survival.

## MATERIALS AND METHODS

### Reagents and antibodies

The small molecule inhibitor of SCD1, {4-(2-chlorophenoxy)-N-[3-(methylcarbamoyl)-phenyl]piperidine-1-carboxamide} (A939572), was purchased from Biofine International (Vancouver, Canada). It was dissolved in DMSO and stored a −20°C.

The bovine serum albumin (BSA)-conjugated oleic acid solution and fatty acid-free BSA were obtained from Sigma-Aldrich (St. Louis, MO, USA).

The “GFP-CertifiedTM Apoptosis/Necrosis Detection Kit” was purchased by ENZO Life Sciences (# ENZ-51002; Farmingdale, NY, USA) and included: staurosporine (STS), that was dissolved in DMSO and stored a −20°C; annexin V-EnzoGold dye and red necrosis stain (7-amino-actinomycin D, 7-AAD).

For Western blot analysis, the following antibodies were used: anti-SCD1 (rabbit polyclonal; Cell Signaling, Danvers, MA, USA), anti-SCD5 (rabbit polyclonal; Aviva Systems Biology, San Diego, CA, USA), anti-β-actin (mouse monoclonal, clone AC-15, Sigma-Aldrich). The secondary antibodies were goat anti-rabbit and horse anti-mouse conjugated to horseradish peroxidase (HRP, Vector Laboratories, Burlingame, CA, USA).

For immunofluorescence, the following antibodies were used: anti-E-cadherin (mouse monoclonal, clone 4A2C7, Thermo Fisher Scientific, Waltham, MA USA) and anti-N-cadherin (mouse monoclonal, clone 32, BD Transduction Laboratories, Lexington, KY, USA). The secondary antibodies were donkey anti-mouse conjugated to Alexa Fluor 488 (for E-cadherin) or to Alexa Fluor 495 (for N-cadherin) and were obtained from Thermo Fisher Scientific.

### Cell cultures

Primary normal and cancer-associated fibroblasts (NFs and CAFs, respectively) were isolated from normal mammary skin and mammary cancer tissue samples and immunocytochemically assayed to validate their fibroblastic nature as previously described [18]. The well-differentiated, poorly invasive, and low metastasizing human breast carcinoma cell line MCF-7 and the highly aggressive, invasive and poorly differentiated MDA-MB-231 human breast carcinoma cells were obtained from American Type Culture Collection (Manassas, VA, USA). All cells were cultured in DMEM (Euroclone, Milan, Italy) supplemented with 10% FBS (Life Technologies, Carlsbad, CA, USA), antibiotics (100 IU/ml penicillin, 100 μg/ml streptomycin, Euroclone) and 2 mM glutamine (Euroclone). Cells were maintained at 37°C in a humidified atmosphere of 5% CO_2_-95% air.

### Co-cultures

In order to assess the effect of fibroblasts (NFs and CAFs) on SCD5 expression in tumor cells, co-cultures of the two cell types were established. Briefly, subconfluent fibroblasts and breast cancer cell cultures were trypsinized and ~5×10^3^/cm^2^ cells of each cell type were plated together in their standard medium in 100 mm Petri dishes. Medium was renewed after 3 days and, after 6 days, the two cell types were separated by magnetic cell sorting, as previously described [19]. MCF-7 and MDA-MB-231 homotypic cultures were used as controls. Co-culture experiments were carried out with fibroblasts (NFs and CAFs) of passage 2-3 to avoid cell aging effects.

### Quantitative real-time PCR

Quantitative real-time PCR (qRT-PCR) analysis was performed in order to investigate the effect produced by fibroblasts on SCD5 mRNA level in breast cancer cells. Briefly, total RNA was extracted from MCF-7 or MDA-MB-231 cells cultured alone or co-cultured with fibroblasts (NFs or CAFs) for 6 days, with the TRIzol RNA Isolation Reagents (Thermo Fisher Scientific). Five μg of RNA were converted into single-stranded DNA by a standard 20-μl RT reaction with the High-Capacity cDNA Reverse Transcription Kit (Thermo Fisher Scientific), according to the manufacturer's instructions. The cDNA obtained from the reverse transcription reaction was amplified by real time PCR with the SensiFast SYBR Hi-ROX kit (Bioline, London, UK) in a total volume of 25 μl. The following cycling condition were used: 95°C for 2 min followed by 40 cycles of 95°C for 5 sec, 60°C for 10 sec, 72°C for 20 sec, on a StepOne Real-Time PCR Systems (Life Technologies). The primers used were as follows, SCD5 (NM_001037582): 5'-ATGTCGTCCTGATGA GCTTG-3' and 5'-CAGGAGGAAGCAGAAGTAGG-3'; β-actin: 5′-ATCTGGCACCACACCTTC-3′ and 5′-AGCC AGGTCCAGACGCA-3′ (IDT Integrated DNA Technologies, Coralville, IA, USA). The level of SCD5 mRNA was expressed as relative-fold change versus the β-actin mRNA. The relative fold change in mRNA expression was calculated using the 2−ΔΔCt method. The mean of ΔCt values for SCD5 amplicon was normalized to that of β-actin and compared with control (MCF-7 or MDA-MB-231 cells cultured alone). Reactions were performed in triplicate.

### Western blot analysis

For SCD1 and SCD5 analysis, tumor cell total protein extracts were obtained in RIPA lysis buffer [50 mM Tris-HCl (pH 7.7), 150 mM NaCl, 1% Triton X-100, 1% sodium deoxycholate, 0.1% SDS] freshly supplemented with phosphatase and protease inhibitors (100 μM Na_3_VO_4_, 0.3 mM phenylmethylsulfonylfluoride, 50 μg/ml leupeptin and 20 μg/ml aprotinin). Thirty μg of total proteins were resolved on a 10% SDS-PAGE and transferred onto an Immobilon P membrane (Millipore, Billerica, MA, USA) which was incubated with the primary antibodies. The blots were then overlaid with a HRP-labelled secondary antibody. The protein bands were detected using an enhanced chemiluminescence system (ECL, GE Healthcare, Piscataway, NJ, USA) and visualised on Hyperfilm ECL (GE Healthcare). β-actin was used as an internal loading control. Band intensities were measured by densitometry (Chemi Doc Documentation System/Quantity One quantitation software, Bio-Rad). Variations in SCD1 and SCD5 protein levels were expressed as fold change compared to control (MCF-7 or MDA-MB-231 cells cultured alone) after normalization to β-actin expression.

### Small interfering RNA

The silencing of SCD1 and SCD5 genes was achieved by using small interfering RNA (siRNA) duplex oligonucleotides against the coding sequence of human SCD1 and SCD5 that were synthesized and purchased by Integrated DNA Technologies (Coralville, IA, USA). For both genes, we selected two specific target sequences as follows: 5'-AAU GAU CAG AAA GAG CCG UAG-3' (sense_SCD1_1) and 5'AAA UCG UCU CCA ACU UAU CUC-3' (sense_SCD1_2); 5'-GGA GAA AGC UUG ACG UCA CUG-3' (sense_SCD5_1) and 5'AAA CUG CCU CUG AGG AUA UUU-3' (sense_SCD5_2). Commercially available non-targeting DSNC1 negative control duplex (Integrated DNA Technologies) was used as experimental control.

MCF-7 and MDA-MB-231 cells were seeded into 6-well plates at a density of 1×10^5^ cells/well and incubated at 37°C until they reached 40% confluence. The cells were then transfected with Opti-MEM Reduced Serum Medium (Thermo Fisher Scientific) containing either 60 pmol of SCD1 or SCD5 siRNA plus Oligofectamine Transfection Reagent (0.3%, v/v; Life Technologies), according to the manufacturer's instructions. Double transfection was performed by transfecting cells with a combination of 60 pmol of both SCD1 and SCD5 siRNA plus Oligofectamine Transfection Reagent (0.3%, v/v). Fresh medium was added 5 h after transfection and cells were further incubated for 72 h prior to proceed with wound healing assay, apoptosis/necrosis assay and/or immunofluorescence. Western blot analysis was performed to determine the efficiency of SCD1 and SCD5 siRNA *in vitro* transfection (Supplementary Figure 1).

### Wound healing assay

To assess the effect of oleic acid on the migration ability of SCD1- or SCD5-silenced tumor cells, a wound healing assay was performed. Forty-eight h following SCD1 or SCD5 siRNA transfection, MCF-7 and MDA-MB-231 cells, grown to subconfluence into 6-well plates, were pretreated for 2 h with mitomycin C (5 μg/ml, Sigma-Aldrich) in serum-free medium in order to block cell proliferation. A scratch was made in each well using a 200 μl pipette tip and the wounded monolayers washed twice with PBS to remove cell debris and floating cells. The following treatments were then added to SCD1- or SCD5-inhibited tumor cells: a) CAF-conditioned medium (CAF-CM), b) 10 μM oleic acid (Sigma-Aldrich), c) CAF-CM plus 10 μM oleic acid.

The wounds (5 randomly selected points per wound) were photographed at 0, 24 and 48 h timepoints under an inverted microscope (100x magnification) with a digital camera and the distance migrated by the cells was measured at the reference points by an image-processing software (ImageJ, version 1.49; imagej.nih.gov/ij/). Three independent experiments were performed with two sets of culture dishes for each condition.

In a further series of experiments performed following the same scheme above described, SCD1 inhibition was obtained also by treating tumor cells for 24 and 48 h with the small molecule inhibitor of SCD1, A939572 (1 μM, Biofine International). The same experimental condition was impossible to realize for SCD5 because of the unavailability of a selective pharmacological inhibitor.

### Immunofluorescence

Seventy-two h following SCD5 siRNA transfection, MCF-7 cells growing on round glass coverslips into 6-well plates were fixed with 4% paraformaldehyde in PBS and permeabilized with 0.2% triton X-100 in 5% BSA containing PBS (PBS/BSA). Cells were washed twice in PBS/BSA and blocked by SuperBlock reagent (UCS Diagnostic, Rome, Italy) for 8 min at room temperature (RT). After washing in PBS/BSA, cells were incubated overnight at 4°C with the primary antibody (anti-E-cadherin or anti-N-cadherin). After three washes, the appropriate fluorochrome-conjugated secondary antibody was added for 30 min at RT, after which slides were washed and mounted using ProLong® Gold antifade reagent containing the blue-fluorescent nuclear dye DAPI (Thermo Fisher Scientific). Negative controls were processed in parallel by omitting primary antibodies.

Images were viewed with an Olympus BX53 fluorescent microscope (Olympus, Tokyo, Japan) and captured with ISCapture software (Tucsen Photonics, Fujian, China).

### Apoptosis/necrosis assay

To investigate the effect of siRNA silencing of SCD5 on MCF-7 cell viability, an Apoptosis/Necrosis Detection Kit (Enzo Life Sciences) was used, according to the manufacturer's instructions with slight modifications. This assay allows to distinguish between cells with early/late apoptotic or necrotic membrane alterations, based on the following criteria: exposure of phosphatidylserine on the extracellular face of the intact plasma membrane of cells in the early apoptosis stage is detected by binding to membrane impermeable annexin V (conjugated to enhanced Cyanine3, Cy3, EnzoGold, yellow emitting). On the other hand, binding of the membrane impermeable DNA intercalating dye 7-AAD (red emitting) to the nuclear DNA indicates membrane damages, typical of necrosis or late stage apoptosis while it is excluded from healthy cells, with intact cell membranes.

Seventy-two h following SCD5 siRNA transfection, MCF-7 cells grown to subconfluence on round glass coverslips into 6-well plates were incubated with a buffer containing annexin V-EnzoGold and the red necrosis stain (apoptosis/necrosis dual detection reagent). Cells were washed, fixed with 2% formaldehyde and the slides mounted using ProLong® Gold antifade reagent containing DAPI (Thermo Fisher Scientific) which stains nuclei.

In another series of experiments, SCD5 siRNA transfected MCF-7 cells were treated with 10 μM oleic acid and the effect on cell viability evaluated after 24 and 48 h.

In all the experiments cells were visualized and counted using an Olympus BX53 fluorescent microscope at 200x magnification (a minimum of 700 cells/field, in 10 random fields were counted) and images captured with ISCapture software (Tucsen Photonics). Results were expressed as the percent of necrotic cells (7-AAD-positive cells) of the total number of counted cells.

Negative controls were obtained by omitting incubation with dual detection reagent. Positive controls were generated by pretreating untransfected MCF-7 cells for 5 h with 2 μM STS, which in Bcl-2 low expressing MCF-7 cells is a necrosis inducer [37].

### Statistical analysis

At least three independent experiments were performed for each protocol. The two-tailed Student's t test was used to compare means of two different groups. Data are expressed as the means ± S.D. Differences between groups were considered significant at p<0.05.

## SUPPLEMENTARY MATERIALS FIGURES AND TABLES



## References

[1] Choe C, Shin YS, Kim SH, Jeon MJ, Choi SJ, Lee J, Kim J (2013). Tumor-stromal interactions with direct cell contacts enhance motility of non-small cell lung cancer cells through the hedgehog signaling pathway. Anticancer Res.

[2] Bhowmick NA, Neilson EG, Moses HL (2004). Stromal fibroblasts in cancer initiation and progression. Nature.

[3] Maxwell PJ, Neisen J, Messenger J, Waugh DJ (2014). Tumor-derived CXCL8 signaling augments stroma-derived CCL2-promoted proliferation and CXCL12-mediated invasion of PTEN-deficient prostate cancer cells. Oncotarget.

[4] Wu X, Tao P, Zhou Q, Li J, Yu Z, Wang X, Li J, Li C, Yan M, Zhu Z, Liu B, Su L (2017). IL-6 secreted by cancer-associated fibroblasts promotes epithelial-mesenchymal transition and metastasis of gastric cancer via JAK2/STAT3 signaling pathway. Oncotarget.

[5] Peetla C, Bhave R, Vijayaraghavalu S, Stine A, Kooijman E, Labhasetwar V (2010). Drug resistance in breast cancer cells: biophysical characterization of and doxorubicin interactions with membrane lipids. Mol Pharm.

[6] Reiss K, Cornelsen I, Husmann M, Gimpl G, Bhakdi S (2011). Unsaturated fatty acids drive disintegrin and metalloproteinase (ADAM)-dependent cell adhesion, proliferation, and migration by modulating membrane fluidity. J Biol Chem.

[7] Tavolari S, Munarini A, Storci G, Laufer S, Chieco P, Guarnieri T (2012). The decrease of cell membrane fluidity by the non-steroidal anti-inflammatory drug Licofelone inhibits epidermal growth factor receptor signalling and triggers apoptosis in HCA-7 colon cancer cells. Cancer Lett.

[8] Sade A, Tunçay S, Cimen I, Severcan F, Banerjee S (2012). Celecoxib reduces fluidity and decreases metastatic potential of colon cancer cell lines irrespective of COX-2 expression. Biosci Rep.

[9] Edmond V, Dufour F, Poiroux G, Shoji K, Malleter M, Fouqué A, Tauzin S, Rimokh R, Sergent O, Penna A, Dupuy A, Levade T, Theret N (2015). Downregulation of ceramide synthase-6 during epithelial-to-mesenchymal transition reduces plasma membrane fluidity and cancer cell motility. Oncogene.

[10] Wang J, Yu L, Schmidt RE, Su C, Huang X, Gould K, Cao G (2005). Characterization of HSCD5, a novel human stearoyl-CoA desaturase unique to primates. Biochem Biophys Res Commun.

[11] Biddinger SB, Almind K, Miyazaki M, Kokkotou E, Ntambi JM, Kahn CR (2005). Effects of diet and genetic background on sterol regulatory element-binding protein-1c, stearoyl-CoA desaturase 1, and the development of the metabolic syndrome. Diabetes.

[12] Igal RA (2011). Roles of StearoylCoA Desaturase-1 in the Regulation of Cancer Cell Growth, Survival and Tumorigenesis. Cancers (Basel).

[13] Li J, Ding SF, Habib NA, Fermor BF, Wood CB, Gilmour RS (1994). Partial characterization of a cDNA for human stearoyl-CoA desaturase and changes in its mRNA expression in some normal and malignant tissues. Int J Cancer.

[14] Thai SF, Allen JW, DeAngelo AB, George MH, Fuscoe JC (2001). Detection of early gene expression changes by differential display in the livers of mice exposed to dichloroacetic acid. Carcinogenesis.

[15] Scaglia N, Igal RA (2005). Stearoyl-CoA desaturase is involved in the control of proliferation, anchorage-independent growth, and survival in human transformed cells. J Biol Chem.

[16] Scaglia N, Chisholm JW, Igal RA (2009). Inhibition of stearoylCoA desaturase-1 inactivates acetyl-CoA carboxylase and impairs proliferation in cancer cells: role of AMPK. PLoS One.

[17] Luyimbazi D, Akcakanat A, McAuliffe PF, Zhang L, Singh G, Gonzalez-Angulo AM, Chen H, Do KA, Zheng Y, Hung MC, Mills GB, Meric-Bernstam F (2010). Rapamycin regulates stearoyl CoA desaturase 1 expression in breast cancer. Mol Cancer Ther.

[18] Angelucci C, Maulucci G, Lama G, Proietti G, Colabianchi A, Papi M, Maiorana A, De Spirito M, Micera A, Balzamino OB, Di Leone A, Masetti R, Sica G (2012). Epithelial-stromal interactions in human breast cancer: effects on adhesion, plasma membrane fluidity and migration speed and directness. PLoS One.

[19] Angelucci C, Maulucci G, Colabianchi A, Iacopino F, D'Alessio A, Maiorana A, Palmieri V, Papi M, De Spirito M, Di Leone A, Masetti R, Sica G (2015). Stearoyl-CoA desaturase 1 and paracrine diffusible signals have a major role in the promotion of breast cancer cell migration induced by cancer-associated fibroblasts. Br J Cancer.

[20] Sinner DI, Kim GJ, Henderson GC, Igal RA (2012). StearoylCoA desaturase-5: a novel regulator of neuronal cell proliferation and differentiation. PLoS One.

[21] Bellenghi M, Puglisi R, Pedini F, De Feo A, Felicetti F, Bottero L, Sangaletti S, Errico MC, Petrini M, Gesumundo C, Denaro M, Felli N, Pasquini L (2015). SCD5-induced oleic acid production reduces melanoma malignancy by intracellular retention of SPARC and cathepsin B. J Pathol.

[22] Shekhar MP, Werdell J, Santner SJ, Pauley RJ, Tait L (2001). Breast stroma plays a dominant regulatory role in breast epithelial growth and differentiation: implications for tumor development and progression. Cancer Res.

[23] Cirri P, Chiarugi P (2012). Cancer-associated-fibroblasts and tumour cells: a diabolic liaison driving cancer progression. Cancer Metastasis Rev.

[24] Mauvoisin D, Charfi C, Lounis AM, Rassart E, Mounier C (2013). Decreasing stearoyl-CoA desaturase-1 expression inhibits β-catenin signaling in breast cancer cells. Cancer Sci.

[25] Scaglia N, Igal R (2008). Inhibition of Stearoyl-CoA Desaturase 1 expression in human lung adenocarcinoma cells impairs tumorigenesis. Int J Oncol.

[26] Roongta UV, Pabalan JG, Wang X, Ryseck RP, Fargnoli J, Henley BJ, Yang WP, Zhu J, Madireddi MT, Lawrence RM, Wong TW, Rupnow BA (2011). Cancer cell dependence on unsaturated fatty acids implicates stearoyl-CoA desaturase as a target for cancer therapy. Mol Cancer Res.

[27] Moon HS, Batirel S, Mantzoros CS (2014). Alpha linolenic acid and oleic acid additively down-regulate malignant potential and positively cross-regulate AMPK/S6 axis in OE19 and OE33 esophageal cancer cells. Metabolism.

[28] Hardy S, Langelier Y, Prentki M (2000). Oleate activates phosphatidylinositol 3-kinase and promotes proliferation and reduces apoptosis of MDA-MB-231 breast cancer cells, whereas palmitate has opposite effects. Cancer Res.

[29] Soto-Guzman A, Navarro-Tito N, Castro-Sanchez L, Martinez-Orozco R, Salazar EP (2010). Oleic acid promotes MMP-9 secretion and invasion in breast cancer cells. Clin Exp Metastasis.

[30] Noto A, Raffa S, De Vitis C, Roscilli G, Malpicci D, Coluccia P, Di Napoli A, Ricci A, Giovagnoli MR, Aurisicchio L, Torrisi MR, Ciliberto G, Mancini R (2013). Stearoyl-CoA desaturase-1 is a key factor for lung cancer-initiating cells. Cell Death Dis.

[31] Chen YZ, Xue JY, Chen CM, Yang BL, Xu QH, Wu F, Liu F, Ye X, Meng X, Liu GY, Shen ZZ, Shao ZM, Wu J (2012). PPAR signaling pathway may be an important predictor of breast cancer response to neoadjuvant chemotherapy. Cancer Chemother Pharmacol.

[32] Radde BN, Alizadeh-Rad N, Price SM, Schultz DJ, Klinge CM (2016). Acid, Salicylic Acid, and Oleic Acid Differentially Alter Cellular Bioenergetic Function in Breast Cancer Cells. J Cell Biochem.

[33] Ariyama H, Kono N, Matsuda S, Inoue T, Arai H (2010). Decrease in membrane phospholipid unsaturation induces unfolded protein response. J Biol Chem.

[34] Schaffer JE (2003). Lipotoxicity: when tissues overeat. Curr Opin Lipidol.

[35] Arouri A, Lauritsen KE, Nielsen HL, Mouritsen OG (2016). Effect of fatty acids on the permeability barrier of model and biological membranes. Chem Phys Lipids.

[36] Borradaile NM, Buhman KK, Listenberger LL, Magee CJ, Morimoto ET, Ory DS, Schaffer JE (2006). A critical role for eukaryotic elongation factor 1A-1 in lipotoxic cell death. Mol Biol Cell.

[37] Poliseno L, Bianchi L, Citti L, Liberatori S, Mariani L, Salvetti A, Evangelista M, Bini L, Pallini V, Rainaldi G (2004). Bcl2-low-expressing MCF7 cells undergo necrosis rather than apoptosis upon staurosporine treatment. Biochem J.

