# Supplementary material for: Pivotal role of human stearoyl-CoA desaturases (SCD1 and 5) in breast cancer progression: oleic acid-based effect of SCD1 on cell migration and a novel pro-cell survival role for SCD5
Source: Oncotarget. 2018 May 11;9(36):24364–80. doi: 10.18632/oncotarget.25273 (PMC5966257; doi:10.18632/oncotarget.25273)
Supplement: Supplementary file 1 [file oncotarget-09-24364-s001.pdf]

Pivotal role of human stearoyl-CoA desaturases (SCD1 and 5) in breast cancer progression: oleic acid-based effect of SCD1 on cell migration and a novel pro-cell survival role for SCD5

SUPPLEMENTARY MATERIALS

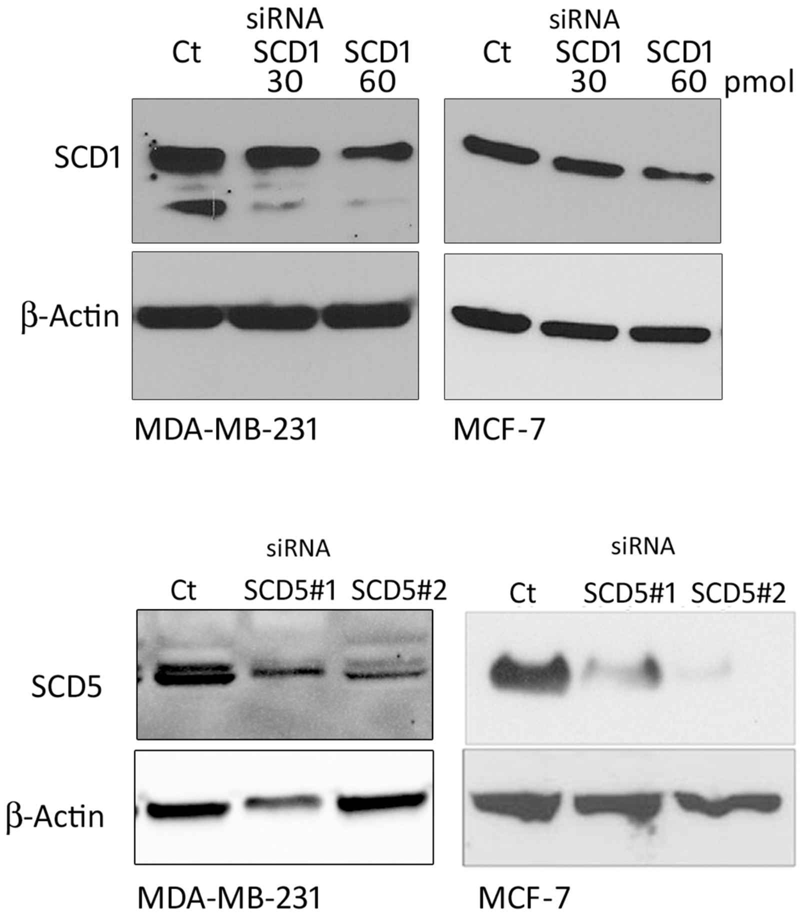

Supplementary Figure 1: Western blot analysis of siRNA-mediated SCD1 and SCD5 knockdown. Indicated doses and sequences of double-stranded RNA moleculescomplementary to the coding sequence of human SCD1 and SCD5 cDNA were used to transiently transfect MCF-7 and MDA-MB-231 cells for 72 h, with Oligofectamine Transfection Reagent (0.3%, v/v). Non-targeting siRNA (control, Ct) was used as a negative control for evaluating RNA interference off-target effects. The silencing efficiency of both siRNAs was evaluated by Western blot analysis using an anti-SCD1 (clone M38, Cell Signaling Technology) and anti-SCD5 (Aviva Systems Biology) antibodies. Protein levels in siRNA-silenced cells were compared to those of controls after normalization to β-actin expression.

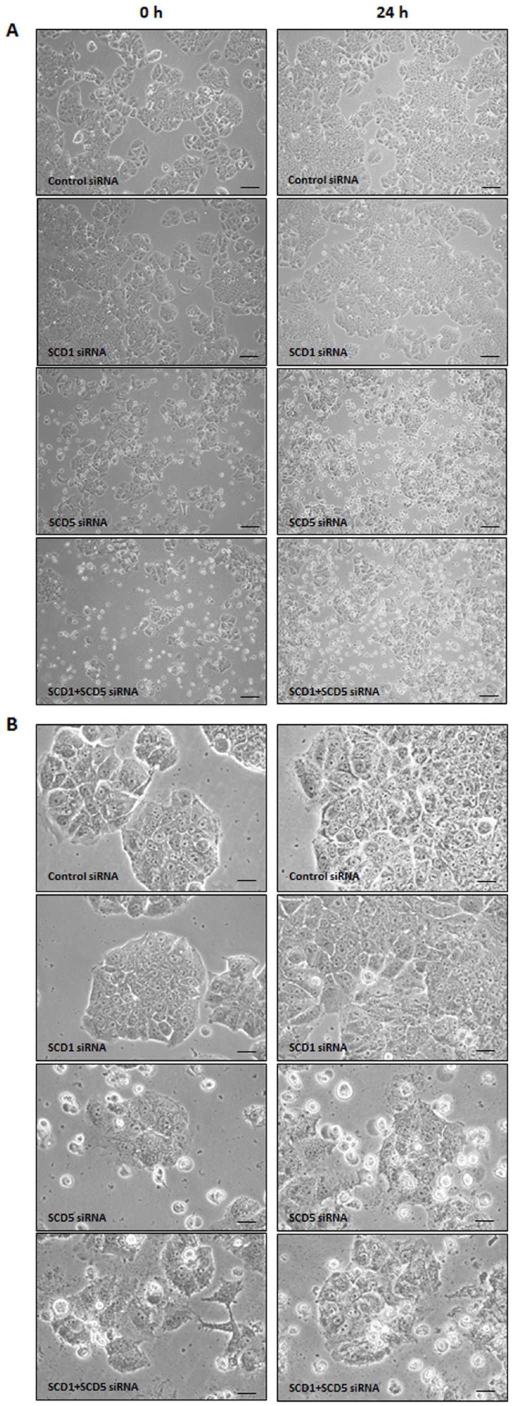

Supplementary Figure 2: Effect of SCD1/SCD5 siRNA double silencing in MCF-7 cells. MCF-7 cells underwent a transient SCD1 or SCD5 knockdown by a 72-h transfection with 60 pmol of SCD1 or SCD5 siRNA oligos plus Oligofectamine Transfection Reagent (0.3%, v/v). SCD1/SCD5 siRNA double silencing was performed by transfecting cells with a combination of 60 pmol of both SCD1 and SCD5 siRNA. The cells grown to subconfluence into 6-well plates were photographed at 0 and 24 h after transfection under an inverted microscope (magnification: A, 100x; B, 400x). Three independent experiments were performed with two sets of culture dishes for each condition. Scale bar = 100 µm.

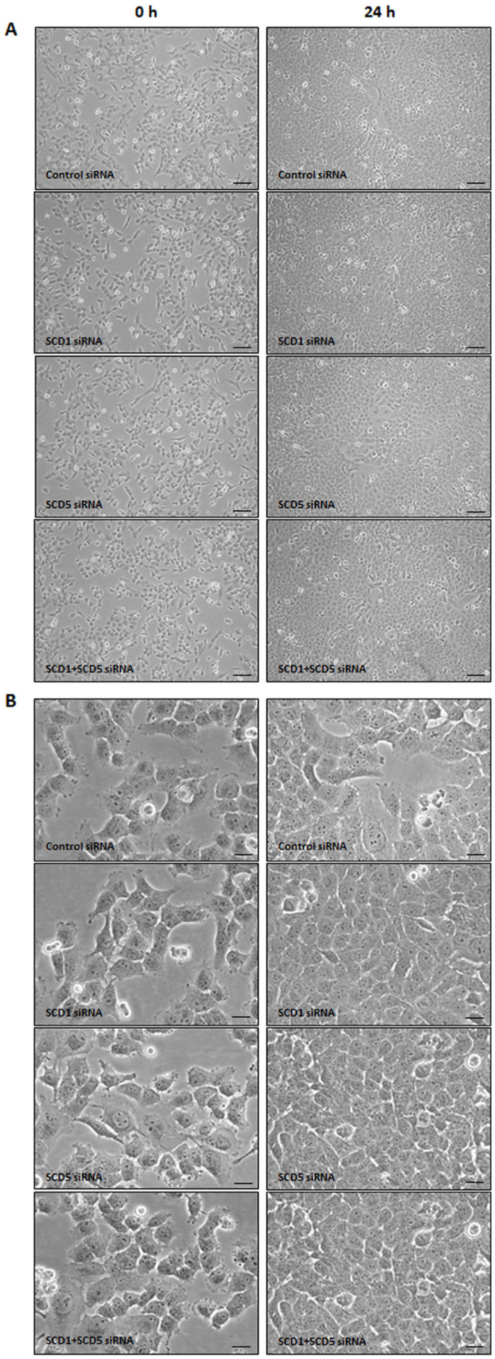

Supplementary Figure 3: Effect of SCD1/SCD5 siRNA double silencing in MDA-MB-231 cells. MDA-MB-231 cells underwent a transient SCD1 or SCD5 knockdown by a 72-h transfection with 60 pmol of SCD1 or SCD5 siRNA oligos plus Oligofectamine Transfection Reagent (0.3%, v/v). SCD1/SCD5 siRNA double silencing was performed by transfecting cells with a combination of 60 pmol of both SCD1 and SCD5 siRNA. The cells grown to subconfluence into 6-well plates were photographed at 0 and 24 h after transfection under an inverted microscope (magnification: A, 100x; B, 400x). Three independent experiments were performed with two sets of culture dishes for each condition. Scale bar = 100 µm.
